# Surfactant Protein D Mediates the Association Between Smoking and Type 2 Diabetes Mellitus Incidence in the Spanish Adult Population: Di@bet.es Study

**DOI:** 10.3390/jox15060184

**Published:** 2025-11-01

**Authors:** Wasima Oualla-Bachiri, Ana Lago-Sampedro, Eva García-Escobar, Cristina Maldonado-Araque, Viyey Doulatram-Gamgaram, Marta García-Vivanco, Fernando Martín-Llorente, Juan Luis Garrido, Elías Delgado, Felipe J. Chaves, Luis Castaño, Alfonso Calle-Pascual, Josep Franch-Nadal, Gabriel Olveira, Sergio Valdés, Gemma Rojo-Martínez

**Affiliations:** 1 Centro de Investigación Biomédica en Red de Diabetes y Enfermedades Metabólicas Asociadas (CIBERDEM), Instituto de Salud Carlos III, 28029 Madrid, Spain; 2 Servicio de Endocrinología y Nutrición, Hospital Regional Universitario de Málaga, IBIMA Plataforma BIONAND, 29590 Malaga, Spain; 3 Facultad de Medicina, Universidad de Málaga, Andalucía Tech, Campus de Teatinos s/n, 29071 Málaga, Spain; 4 División De Contaminación Atmosférica, Centro de Investigaciones Energéticas, Medioambientales y Tecnológicas (CIEMAT), 28040 Madrid, Spain; 5 Centro de Investigación Biomédica en Red de Enfermedades Raras (CIBERER), Instituto De Salud Carlos III, 28029 Madrid, Spain; 6 Department of Endocrinology and Nutrition, Central University Hospital of Asturias, Health Research Institute of the Principality of Asturias (ISPA), University of Oviedo, 33011 Oviedo, Spain; 7 Genomic and Genetic Diagnosis Unit, INCLIVA Biomedical Research Institute, 46010 Valencia, Spain; 8 Cruces University Hospital, Bio-Bizkaia, Department of Pediatrics, University of the Basque Country (UPV/EHU), European Reference Network on Rare Endocrine Conditions (Endo-ERN), 48903 Barakaldo, Spain; 9 Department of Endocrinology and Nutrition, San Carlos University Hospital of Madrid, 28040 Madrid, Spain; 10 EAP Raval Sud, Catalan Institute of Health, GEDAPS Network, Primary Care, Research Support Unit (IDIAP-Jordi Gol Foundation), 08007 Barcelona, Spain

**Keywords:** type 2 diabetes mellitus, incidence, SP-D, xenobiotics, smoking, air pollutants, biomarkers, mediation

## Abstract

It is well known that environmental factors influence the risk of type 2 diabetes mellitus (T2DM). Several studies have linked the xenobiotics present in tobacco or air pollutants to T2DM development, although the underlying mechanisms remain unclear. Surfactant protein D (SP-D), an immune component released into the bloodstream after lung injury, has been associated with metabolic diseases. The aim of this study was to investigate whether SP-D mediates the effects of smoking or air pollution exposure on T2DM risk in the Spanish adult population. Socio-demographic, lifestyle (including smoking status) and clinical data from 2155 participants from the Di@bet.es cohort were analyzed. Annual concentrations of PM_10_, PM_2.5_, SO_2_, CO and NO_2_ according to participants’ residential address codes were used to study air pollution exposure. T2DM was diagnosed at baseline and after 7.5 years of follow-up. SP-D serum levels were measured by ELISA and categorized as above or below the 25th percentile. Our results revealed a higher percentage of smokers in the high SP-D category; however, no associations were observed between air pollutants (PM_10_, PM_2.5_, SO_2_, CO) and SP-D categories. Both smoking and elevated SP-D levels were found to increase the risk of T2DM independently. Mediation analysis indicated that SP-D mediates 14% of the effect of smoking on T2DM incidence in the Spanish adult population.

## 1. Introduction

Around 588.7 million people worldwide have diabetes currently, and if trends continue the number will jump to an estimate of 852.5 million by 2050, which means a 45% increase. Type 2 diabetes mellitus (T2DM), accounting for over 90% of all diabetes cases, is a major public health concern [1]. While genetic susceptibility and lifestyle factors such as diet and physical inactivity are well-known contributors to the development of T2DM [2,3], emerging evidence highlights the significant role of environmental exposure in the pathogenesis and progression of diabetes [4,5]. Among these environmental factors, exposure to xenobiotics—chemical substances that are foreign to an organism—has received increasing attention due to their potential to influence T2DM and related comorbidities, such as obesity, non-alcoholic fatty liver disease and cardiovascular disease [6].

Tobacco contains a complex mixture of xenobiotics. The total number of chemical compounds identified in tobacco plus tobacco smoke reached 9582, of which 6010 were identified in tobacco smoke alone [7]. Similarly, airborne particulate matter (PM), such as PM_10_ and particularly PM_2.5_, carries various xenobiotics (volatile organic compounds, heavy metals, polycyclic aromatic hydrocarbons, etc.) that can enter the body through respiration and trigger proinflammatory responses [8]. SO_2_, CO and NO_2_ gases, originating from the combustion of fossil fuels, are also foreign chemical substances [9]. Several studies have demonstrated that both tobacco and air pollution are associated with an elevated risk of type 2 diabetes and its complications. Smoking has been linked to a 30–40% increased risk of T2DM for active smokers compared to non-smokers [10]. Cigarette smoking has been associated with an increased risk of T2DM in Japan, with a linear dose–response relationship between consumption and risk [11]. Ambient air pollution exposure may also contribute to increased risk of incidence and progression of T2DM [12]. Long-term exposure to ambient SO_2_ was related with a higher risk of developing T2DM in the Taiwanese population as reported by Li et al. [13]. Exposure to high levels of PM_10_, SO_2_ and NO_2_ are associated with an elevated risk of T2DM incidence in China [14]. An increase in ambient PM_2.5_ levels was associated with increased odds of hypertension and T2DM [15]. Furthermore, several meta-analyses have supported the increased risk of T2DM incidence in relation with air contaminants exposure [16,17,18].

Detecting early indicators that link environmental exposure with T2DM remains necessary in order to understand the interplay between the environment and the disease. Surfactant protein D (SP-D) is a component of the innate immune system that is secreted as part of pulmonary surfactant. It plays a crucial role in defending against inhaled microorganisms, contaminant particles, toxins or allergens. As SP-D migrates from the alveolar epithelium to the bloodstream after lung damage, it has been proposed in several works as a biomarker for lung diseases [19]. However, evidence also suggests that SP-D may also be linked to metabolic diseases such as obesity or T2DM [20]. The underlying mechanisms that mediate the effects of SP-D on these diseases remain unclear, although inflammation may be in the crossroad [21].

We hypothesized that lung damage resulting from smoking or air contaminant exposure could cause an increase in serum SP-D levels, which could be related to the onset of T2DM. The aim of this study was to assess whether SP-D could act as a mediator between smoking or air contaminant exposure and T2DM development in the general Spanish adult population.

## 2. Materials and Methods

### 2.1. Study Population

**Figure 1:**
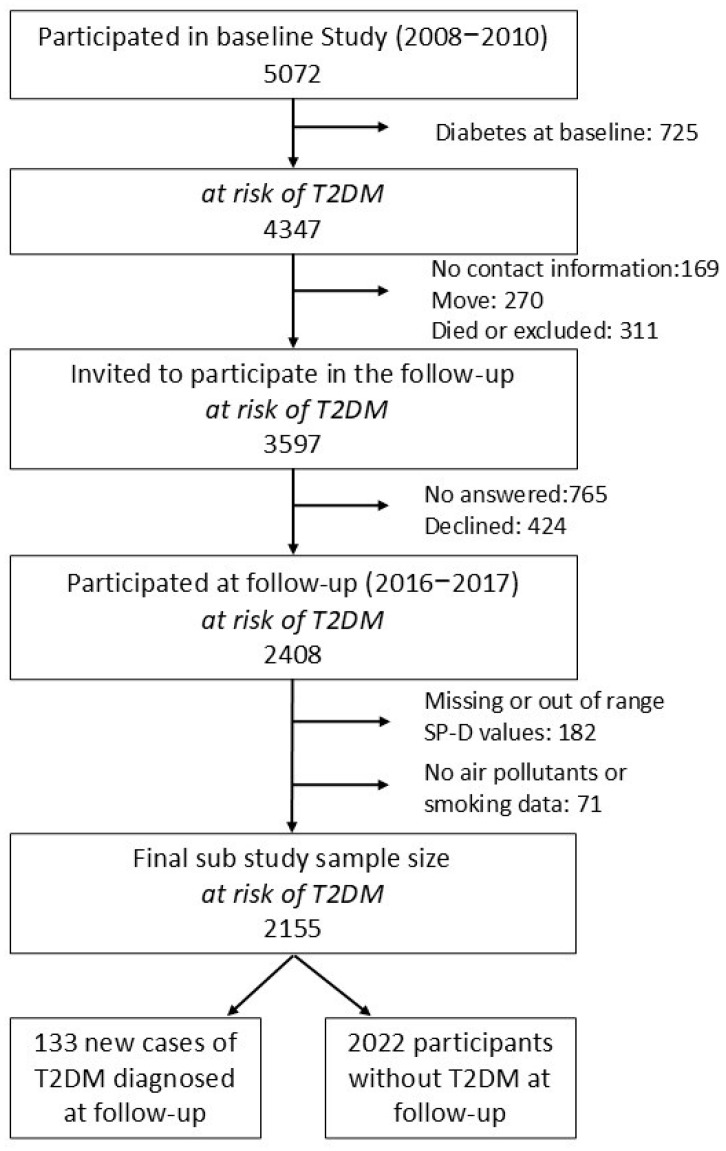
Flow diagram of the study population. T2DM: type 2 diabetes mellitus.

The study population was based on the Di@bet.es study. Detailed information on the Di@bet.es study has been previously described [22,23], and the participant flow chart is presented in Figure 1. In brief, the Di@bet.es epidemiological trial was a population-based cohort study conducted between 2008 and 2010, with a re-evaluation between 2016 and 2017 (with an average follow-up time of 7.5 ± 0.6 years). The initial aim of this study was to evaluate the epidemiology of T2DM in Spain. Participants, aged over 18 years, were selected from the National Health System registries distributed into 100 clusters (primary healthcare centers) using a cluster sampling design, in order to form a representative sample of the Spanish general adult population. Exclusion criteria included institutionalization, pregnancy or breastfeeding, serious illness or surgery that prevented participation in the study or not signing the informed consent form. The final baseline sample comprised 5072 individuals, of whom 2408 participated in the follow-up. Written informed consent was obtained from all participants. In both the cross-sectional and follow-up studies, the surveys and physical examinations were performed by specially trained health personnel who traveled to the recruitment centers to conduct the study under the supervision of a national steering committee and a single coordinator.

For our sub-study, only followed-up participants without T2DM at baseline and with no missing data on serum SP-D levels, air pollutants exposure and tobacco use were selected. Final study sub-population resulted in 2155 followed-up participants at risk of T2DM. A detailed participant flow chart is provided in Figure 1.

The study was approved by the local ethics and clinical investigation committee, as well as by other regional ethics and clinical investigation committees all over Spain. The research was carried out in accordance with The Code of Ethics of the World Medical Association’s Declaration of Helsinki for experiments involving humans [24].

### 2.2. Variables and Procedures

Anthropological and sociological data were collected using a structured questionnaire administered by an interviewer. Physical examinations were performed and blood samples collected after overnight fasting. All samples were analyzed in the same central laboratory after shipment through a specialized courier (Cerba International Laboratory in Barcelona using the Mean Architect C8000 system, Abbott Laboratories, Madrid, Spain) in the cross-sectional study and in the General Laboratory of the Hospital Regional Universitario of Malaga using the Mean ADVIA Chemistry Systems Assays (Siemens Healthineers, Madrid, Spain in the follow-up).

The following variables were considered in this study: age, weight, height, fasting plasma glucose, total cholesterol, LDL cholesterol, HDL cholesterol, triglycerides, systolic blood pressure and diastolic blood pressure. Sex was defined as men/women (self-reported in the questionnaire). BMI was calculated as weight/height^2^ (kg/m^2^). Insulin resistance was estimated using the homeostasis model assessment (HOMA) [25], and the insulin resistance risk category (HOMA-IR) was calculated as the HOMA 75th percentile of our population excluding subjects with T2DM. Family history of T2DM was recorded; the presence of T2DM at baseline was defined as patients requiring oral antidiabetic drugs/insulin therapy and diagnosed prior to admission. Regarding exposure variables, smoking status/habit was defined as current smokers vs. former/never been smokers. Tobacco use/consumption/exposure referred exclusively to smoked tobacco in this study. Total cigarettes variable was calculated by multiplying the number of cigarettes per day by the duration of the smoking habit up to the study baseline, for both current and former smokers. The modeled mean annual concentrations of PM_10_, PM_2.5_, NO_2_, CO and SO_2_ in Spain for the period 2008–2010 were calculated using the CHIMERE chemistry-transport model [26]. This model calculates the concentrations of gaseous species, as well as both inorganic and organic aerosols of primary and secondary origin. These include primary particulate matter, mineral dust, sulphate, nitrate, ammonium, secondary organic species and water. The model has undergone extensive evaluation in Spain through comparison with measured air pollutants at numerous monitoring sites [27,28]. The model was applied to a domain covering the Iberian Peninsula with a horizontal resolution of 0.1 × 0.1°, and the modeled concentrations were corrected using observed values, according to a methodology described by Martín et al. [29]. The average annual exposure to air pollutants for each participant was assigned by interpolating the estimated concentrations to the centroid of their residential address in the year of their health examination. New cases of T2DM at follow-up were diagnosed as fasting serum glucose equal to or higher than 126 mg/dL or 2 h post OGTT equal to or higher than 200 mg/dL or HbA1c equal to or higher than 6.5% or use of glucose-lowering medication at the follow-up examination [30].

Serum SP-D levels were measured by ELISA (Human SP-D ELISA Kit EH3809; FineTest) according to the manufacturer’s instructions. Serum samples were diluted 1:2. The assay had a sensitivity of 0.938 ng/mL and a range of 1.563–100 ng/mL. Those values which were out of range were excluded. Serum levels of SP-D were analyzed as a quantitative variable to assess association with environmental exposure variables and categorized according to the 25th percentile (low and high categories) for descriptive purposes and for association analyses related to T2DM development.

### 2.3. Statistical Analysis

All statistical analyses were performed by using R Statistical Software (version 4.4.3) for Windows, and a *p* value ≤ 0.05 was considered to be significant. Normal distribution of the variables was assessed with the Lillie test, Q–Q plots and histograms. Those variables that were not normally distributed were normalized by using ordered quantile (ORQ) normalization with the Bestnormalize package. Homogeneity of the variances was tested using Levene’s test. Descriptive results are expressed as median (interquartile range) or N (percentage of subjects). Adjusted ANOVA or χ^2^ Pearson test were performed to assess the differences between variables. Clinical criteria were used to select the confounder variables. Spearman partial correlation tests were used to test the relationship between SP-D and exposure variables while controlling for age and sex. T2DM incidence was evaluated by using generalized regression models adjusted by potential confounders. Causal mediation analyses were performed using the Mediation package to assess the indirect effect of tobacco use on T2DM incidence through SP-D. Total, direct and indirect effects were estimated, and quasi-Bayesian confidence intervals were used to evaluate statistical significance. Adjustment for confounding variables (age, sex, BMI, fasting serum glucose, family history of T2DM, insulin resistance) was performed in this analysis. To represent the hypothesized causal framework and clarify potential confounding, we constructed a directed acyclic graph (DAG).

## 3. Results

### 3.1. Baseline Characteristics of the Population at Risk of T2DM According to SP-D Categories

The study sample included 2155 individuals with a mean age of 48 years (age range 18–89 years), of whom 61.1% were women. SP-D was first categorized in quartiles to calculate the incidence of diabetes in each category (Q_1_: 3.42%; Q_2_: 6.28%; Q_3_: 7.14%; Q_4_: 8.30%, *p* < 0.01). Then, the 25th percentile of SP-D was used to define the low SP-D and high SP-D categories (low: 1.56–6.93 ng/mL, n = 587; high: 6.93–95.86 ng/mL, n = 1568) due to an increase in the incidence of T2DM observed at this cut-off point.

Differences in baseline characteristics according to the SP-D categories are presented in Table 1, adjusted by age, sex and BMI, as appropriate. Subjects in the high SP-D category had higher levels of fasting serum glucose, triglycerides, systolic blood pressure and diastolic blood pressure. Additionally, lower HDL cholesterol levels were observed in individuals included in the high SP-D category.

No associations were found when BMI, insulin resistance, total cholesterol and LDL cholesterol levels were examined across SP-D categories. However, there were sex differences regarding the percentage of men and women in each category. The percentage of men increased in the high SP-D category, whereas the percentage of women decreased. Regarding exposure factors, a higher percentage of smokers were found among subjects with high SP-D levels (29.7% vs. 15.2%, *p* < 0.001). No significant associations were found regarding SP-D categories and average exposure to PM_10_ and PM_2.5_. A similar result was found when CO and SO_2_ contaminant gases were studied. Only significant differences were found when NO_2_ was compared between SP-D categories.

### 3.2. Association Between Serum SP-D Levels and Environmental Exposure Variables

Significant correlations were found between serum SP-D levels and tobacco exposure variables (Table 2). These results showed a substantial positive correlation between serum SP-D levels and both the duration of tobacco use in years and the total number of cigarettes consumed prior to the start of this study. When air pollutants exposure and serum SP-D levels were examined, only weak but significant correlations were found with NO_2_ levels in our study population.

### 3.3. T2DM Incidence After 7.5 Years According to SP-D Categories

The proportions of subjects who developed T2DM according to SP-D categories were studied. In our population, 133 (6.17%) people developed T2DM after 7.5 years of follow-up; of these, 21 were in the low SP-D category while 112 were in the high SP-D category. A significant difference was found between the cumulative incidence of diabetes in the low SP-D category vs. the high SP-D category (3.58% vs. 7.14%, *p* < 0.01 measured by χ^2^ test).

When a sex-based stratification analysis was performed, a higher incidence of diabetes was found in the high SP-D category for both sexes (men: 4.21% vs. 8.71, *p* = 0.09; women: 3.27 vs. 6.41%, *p* = 0.03), reaching significance only in women.

Results for the association analysis between SP-D categories and incident T2DM, as determined by adjusted multivariate generalized linear models, are shown in Table 3. Those subjects who had SP-D levels ranged over 25th percentile had almost two times more likelihood of developing T2DM than those who had SP-D levels below this threshold. In our model, smokers have 62% higher odds of developing T2DM compared to former/never smokers.

Multivariable analyses of the risk of T2DM considering NO_2_ were also performed with non-significant results and having no effect on the association between SP-D categories and T2DM development. Additionally, the possible interactive effects of air pollutants and smoking in the new onset of T2DM were evaluated by including the interaction factor “each pollutant*smoking” in the models, returning no significant association. 

### 3.4. SP-D as a Mediator Between Smoking and T2DM

**Figure 2:**
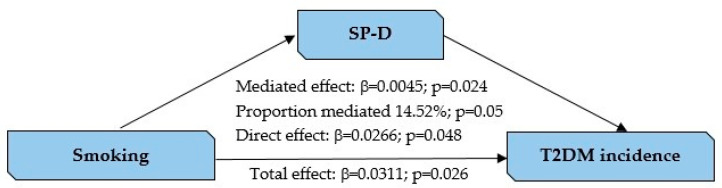
Conceptual and statistical diagram of mediation model. β was calculated with Mediation package in R studio (version 4.4.3). SP-D categories were defined as low vs. high based on 25th percentile. Smoking was defined as current vs. former/never smoker. T2DM incidence was defined as yes vs. no after follow-up.

The causal mediation analysis revealed significant effects of smoking on T2DM incidence, mediated by SP-D. The total effect of smoking on T2DM incidence was estimated to be 0.0311 (95% CI: 0.0042 to 0.06, *p* = 0.026), indicating that smoking was associated with a significantly increased risk of T2DM. The mediation effect (indirect effect) of SP-D was positive and statistically significant, with an estimate of 0.0045 (95% CI: 0.0009 to 0.01, *p* = 0.024), suggesting that SP-D partially mediates the relationship between smoking and T2DM incidence. The direct effect of smoking on T2DM incidence, independent of SP-D, was also significant, with an estimate of 0.0266 (95% CI: 0.0011 to 0.06, *p* = 0.048). The proportion of the total effect mediated by SP-D was 0.1452 (95% CI: 0.0027 to 0.55, *p* = 0.05), indicating that approximately 14.52% of the association between smoking and T2DM incidence was explained by SP-D. These findings emphasize the significant role of SP-D as a mediator in the pathway linking smoking to an increased risk of T2DM (Figure 2).

## 4. Discussion

The role of serum SP-D is still debated, and the precise mechanisms linking SP-D to metabolic disturbances—particularly type 2 diabetes mellitus (T2DM)—have yet to be fully elucidated [31]. To the best of our knowledge, this is the first study to evaluate the association between SP-D and incident T2DM after 7.5 years in a representative Spanish adult population. Our findings suggest that subjects with elevated SP-D levels at baseline are more likely to develop T2DM than those with low SP-D levels, independent of traditional T2DM risk factors such as age, baseline fasting serum glucose level or BMI, among others.

Many studies have quantified human serum SP-D levels, especially to investigate pulmonary diseases, although they differ in methodology and reported values. However, reference values have not yet been established, and SP-D levels measurement is not implemented in clinical practice. It is well known that SP-D levels can be affected by individual or population characteristics. Thus, Sorensen et al. reported serum SP-D levels with a median value of 913 ng/mL (10th percentile: 512 ng/mL; 90th percentile: 1868 ng/mL) [32] when quantification was performed using their own previously described immunoassay [33]. When the BioVendor R&D ELISA kit was used to detect SP-D in Spanish studies, similar range values were found among them (mean values in normoglycemic subjects of 71.9 ng/mL [20] or 97.6 ng/mL [34]). In our study, serum SP-D levels ranged from 1.56 ng/mL to 95.86 ng/mL and were measured using the FineTest ELISA kit. To our knowledge, no previous studies have measured SP-D levels with the same kit, although our studied values were inside the range of detection of the kit.

Various studies have examined the relationship between SP-D and the presence of metabolic disturbances such as obesity, T2DM or insulin resistance, yielding different results. Circulating SP-D levels were inversely associated with BMI in the Danish population-based study GEMINAKAR, which suggested an association between low circulating SP-D and obesity [32]. However, no differences were found in relation to BMI in our study. This different result could be due to the selected population, as Danish twins without T2DM and cardiovascular disease comprised the GEMINAKAR study population, while our study population was the general Spanish population without T2DM. Nevertheless, similar results were found in our study regarding differences in SP-D categories across age, sex and smoking when compared with the GEMINAKAR study, as SP-D levels increase with age and are higher in men and smokers. Fernández-Real et al. found that serum SP-D values were lower among patients with T2DM in a Caucasian cohort of 388 subjects from northern Spain, which was additionally related to decreased insulin sensitivity [20]. In our study, no differences were found regarding SP-D categories and insulin resistance. Although both study populations are Spanish, the difference in the number of subjects studied (388 vs. 2155) and the geographical area covered (North vs. whole Spain) could explain our different results. When fasting serum glucose levels were studied, we found higher glucose levels in those subjects who were included in the high SP-D category. This would be in line with previous results from a case–control study in which López-Cano et al. reported that Spanish individuals with obesity and T2DM exhibited higher serum SP-D concentrations than control subjects [34].

As far as we know, only one previous study has evaluated the impact of SP-D on T2DM incidence. The Tanno–Sobetsu study examined the impact of various lung injury biomarkers on T2DM development. Their results show that 3.1% of the patients developed T2DM during the three-year follow-up, but no relationship was found with SP-D levels when pulmonary proteins were studied in the Japanese population [35]. In our study, an incidence of 6.7% was found, and serum SP-D levels were associated with an increased likelihood of developing T2DM. Our longer follow-up period (7.5 years) and higher sample size could explain the higher incidence rate and the associations with SP-D categories found in our population. Furthermore, there is a possibility that variations in these results may be associated with ethnicity.

The mechanism underlying the relationship between lung injury and T2DM development is still uncertain. Some authors have reported a deleterious effect of T2DM on pulmonary function which could influence serum SP-D levels [34]. Others have reported that inflammation of viscera and other tissues precedes diabetes [35]. Regarding inflammatory biomarkers, elevated proinflammatory cytokines in plasma such as TNF-α could influence the development of diabetes [36]. It has been reported that circulating SP-D induces TNF-α secretion in monocytes through osteoclast-associated receptor signaling (OSCAR) in mice, although the molecular mechanism in humans has not yet been proved [37]. In humans, insulinase activity has been shown to be inversely associated with SP-D levels [20], which could be a possible mechanism involved in the association between SP-D and glucose metabolism, although evidence remains scarce. Apart from the respiratory system, evidence shows that SP-D is expressed in a broad range of tissues and organs such as the brain, liver or vascular endothelium or uterus [19,38,39,40], and this is due to its immune implications as a pattern recognizer molecule, which could influence the origin of serum SP-D and its molecular interactions. As our study found an association between SP-D and incident T2DM, our results support the idea that low-grade inflammation may precede the development of diabetes.

Regarding the exposure factors that could affect SP-D levels, smoking and pollution, considered as direct lung injury agents, were studied. We found a significant association between smoking and SP-D categories, with a higher percentage of smokers in the high SP-D category. This finding is consistent with previous literature supporting that cigarette smoking mediates translocation of SP-D from the lungs to the blood, resulting in increased levels of serum SP-D in smokers compared to non-smokers [41]. Furthermore, our study reveals a significant positive correlation between the number of cigarettes smoked per day, the duration of the smoking habit in years and the total cigarettes consumed with serum SP-D levels, which has not been previously broadly studied in the general population so far. Nevertheless, it is well established that the risk of developing cardiovascular disease, cancer and respiratory conditions is strongly associated with both the number of cigarettes smoked per day and, more importantly, the duration of smoking [42]. Additionally, a potential dose-dependent effect of smoking on the development of T2DM has been reported: smoking cessation is associated with a reduced risk of diabetes, although both current smokers and recent quitters have an elevated diabetes risk proportional to lifetime tobacco exposure, as described in a meta-analyses by Park et al. [43]. SP-D could be suggested to be a proxy biomarker for diabetes risk in relation to smoking habit, helping to identify smoking sub-populations at high risk of diabetes. These findings also highlight the importance of tobacco consumption reduction or cessation to minimize the deleterious effect of smoking not only over lung function but also on diabetes risk [44].

When it comes to air pollution exposure, a meta-analysis consisting of 10 studies with 1985 subjects revealed that PM exposure was associated with a significant reduction in circulating SP-D [45]. However, we have not found significant differences between PM_10_, PM_2.5_, SO_2_, CO and SP-D categories in our study population, and no significative correlations with serum SP-D levels were found. This may be because substantial reductions in ambient concentration levels for all the pollutants studied were recorded in Spain between 1993 and 2017, which covers the period before and during our study [46], and no extreme differences or “out of normative” air contaminant exposure were found in this period. The absence of regions with high exposure could explain why we have not identified significant differences. The Spain Air Quality Evaluation Report in 2016 revealed that the normative annual limit values of air pollutants exposure have mostly been complied in Spanish territory for PM_10_ (<40 μg/m^3^), PM_2.5_ (<25 μg/m^3^), CO (10mg/m^3^) and NO_2_ (<40 μg/m^3^) and the daily limit value for SO_2_ (<125 μg/m^3^ no more than three times) [47]. Regarding NO_2_, as far as we reviewed, no previous studies have described the association between NO_2_ and serum SP-D levels. We found significant differences between NO_2_ levels across SP-D categories and a slight correlation with serum SP-D levels, although NO_2_ levels were not associated with incident T2DM in our study population. Although synergistic action of air pollutants exposure and smoking could jointly trigger inflammatory activity involved in the genesis of T2DM, the interactive effect was not significant in our analyses. Limited incident cases of T2DM and adjustment for multiple risk factors complicated the interaction analyses. These are the reasons why we decided to focus on the study of SP-D as a mediator between smoking and T2DM incidence.

Finally, we demonstrate that SP-D could partially mediate the effect of smoking on T2DM development, particularly 14% of the final effect in our study population. It is recognized that smoking has a diabetogenic effect and impacts on carbohydrate metabolism [42]. Although several mechanisms involved in this association have been proposed, understanding the pathophysiology of it remains complex. Tobacco use has been linked to impaired glucose regulation and increased insulin resistance [48], both of which are key pathways leading to T2DM development. The presence of insulin resistance in smokers can be explained by both the direct and indirect effects of nicotine [49]. Nicotine is involved in central fat accumulation [50] and IRS-1 Ser636 phosphorylation, which impairs insulin signaling and reduces insulin sensitivity [51]. Smoking induces chronic inflammation and oxidative stress, which can contribute to β-cell dysregulation [52]. However, no studies regarding the molecular role of SP-D in the interplay between smoking and T2DM development have been reported, and further research is needed to understand the underlying pathways involved, although this study shows that a significant SP-D mediated mechanism could exist.

Although the statistical significance seems to be modest, the magnitude of the observed effects of SP-D categories regarding T2DM development are nearly double, and the proportion of mediation of SP-D between smoking and incident T2DM is considerable. The clinical relevance of our findings emphasizes the importance of the immune system in the development of diabetes and provides insight into the possible directionality of the associations in explaining the risk of incident T2DM. Given that smoking is a modifiable external factor, these findings reinforce the need for preventive interventions aimed at reducing tobacco consumption as a strategy not only to decrease pulmonary damage but also to lower the risk of developing T2DM. Furthermore, SP-D could become established as an early indicator of systemic inflammation and metabolic dysfunction associated with smoking, enabling the identification of individuals at higher risk and facilitating the implementation of personalized preventive measures. However, further research is needed to validate these results, establish reference values and elucidate the pathophysiological mechanisms underlying these associations before SP-D can be adopted in routine clinical risk assessment.

The main strength of this research is that data were obtained from a sizable nationwide cohort, with a considerable follow-up duration and a substantial number of events (133 incident cases of T2DM). Most participants underwent an OGTT for T2DM diagnosis, ensuring the capture of most incident T2DM cases. All procedures were performed by the same trained staff, both at baseline and during follow-up, so data and sample collection are expected to be highly consistent. We have also included extensive individual-level data including clinical and lifestyle variables, which enabled us to perform a robust multivariate adjustment of the data. Finally, our nationwide perspective enables us to extrapolate our results more widely than local or regional studies, which increases the implications of the findings for the public health system.

However, our study also presents some limitations. It is not possible to know what portion of total serum SP-D levels comes from lungs and what portion comes from other tissues, since it has been reported that many tissues secrete SP-D [19]. Currently, there are no established reference ranges for serum SP-D, and its assessment is not standardized or routinely used in clinical settings, which contributes to variability in measurements depending on the population studied and the techniques employed. Another limitation is that we did not study SP-D levels at follow-up, which could reveal whether subjects who developed T2DM after follow-up had increased serum SP-D levels compared to their baseline levels. Additionally, we used ambient outdoor pollutant levels modeled at the residential addresses of participants as a proxy for air pollution exposure, since no other relevant data—such as time–activity patterns, proximity to major roads, occupational exposures, personal monitoring information or mobility of the participants—were available. Using modeled pollutant concentrations may introduce exposure measurement error, which could have attenuated our effect estimates. However, this is a common limitation in most studies assessing the health impacts of air pollution. In fact, air quality guidelines primarily focus on ambient (outdoor) air pollution for their recommendations [53]. Moreover, our exposure models were based on annual pollutant exposures, as more refined measures to examine different lag periods were not available. Regarding cigarette smoke, we have not evaluated the individual contribution of each chemical component present in tobacco smoke and therefore did not discuss their impact, which would require dedicated analytical approaches beyond the scope of the present study. Other limitations of our study include the observational nature of this study, meaning that we cannot establish causal associations or exclude residual confounding factors in the relationship between air pollutants, tobacco use and SP-D levels; however, the results of our mediation analysis allow us to elucidate the possible directionality of this relationship on the explanation of the risk of incident T2DM at follow-up. Experimental designs or more advanced methods of causal inference are required for establishing it with greater certainty. Finally, given that multiple risk factors were examined, the possibility of false-positive associations cannot be excluded. While our analyses were guided by predefined hypotheses, some of the observed associations were modest in strength and should therefore be interpreted with caution. Replication of these findings in independent populations will be important to confirm the robustness of the observed relationships.

## 5. Conclusions

In conclusion, our study of a representative Spanish cohort demonstrated that high SP-D levels are associated with an increased risk of developing T2DM. Our findings have also pointed out that smoking is a risk factor for the development of diabetes in our study population and SP-D acts as a mediator in this association. Altogether, our results support the recommendations about smoking cessation to reduce the risk of T2DM. Further investigations are needed to reveal the underlying molecular mechanisms and to confirm these associations.

## Figures and Tables

**Table 1:** Baseline general characteristics of the study population ^1^.

Variable	Overall Population (n = 2155)	Low SP-D (n = 587)	High SP-D (n = 1568)	*p* Value
Age, years	48.00 (37.00–60.00)	45.00 (36.00–57.00)	48.00 (37.00–60.00)	*p* < 0.01
Sex, N (%)				
Men	838 (38.9%)	190 (32.4%)	648 (41.3%)	*p* < 0.001
Women	1317 (61.1%)	397 (67.6%)	920 (58.7%)	
BMI, kg/m^2^	27.10 (24.18–30.18)	27.07 (24.13–29.88)	27.11 (24.21–30.31)	0.32
Fasting serum glucose, mg/dL	91.80 (84.24–99.36)	91.26 (83.16–98.28)	92.16 (84.87–99.54)	0.03
HOMA	1.63 (1.14–2.41)	1.59 (1.15–2.41)	1.63 (1.13–2.41)	0.76
Total cholesterol, mg/dL	197.22 (171.11–222.35)	194.90 (167.83–219.64)	197.60 (172.47–223.12)	0.22
LDL cholesterol, mg/dL	104.80 (84.69–125.29)	102.86 (83.14–123.74)	105.18 (85.07–125.68)	0.13
HDL cholesterol, mg/dL	51.82 (44.47–60.71)	52.59 (45.24–61.48)	51.43 (43.70–60.71)	0.02
Triglycerides, mg/dL	97.43 (72.63–132.86)	96.55 (69.97–129.32)	98.32 (74.40–133.75)	*p* < 0.01
Systolic blood Pressure, mmHg	126.50 (115.00–140.00)	124.33 (112.00–138.00)	127.50 (115.50–140.50)	*p* < 0.001
Diastolic blood Pressure, mmHg	75.50 (69.00–82.50)	74.50 (68.50–81.67)	76.00 (69.33–83.00)	0.02
Smoking habit, N (%)				
Smokers	555 (25.8%)	89 (15.2%)	466 (29.7%)	*p* < 0.001
Former/never smokers	1600 (74.2%)	498 (84.8%)	1102 (70.3%)	
Family history of T2DM, N (%)	1069 (49.6%)	297 (50.6%)	772 (49.2%)	0.61
Insulin resistance, N (%)	463 (22.3%)	126 (22.4%)	337 (22.3%)	1.00
PM_10_ exposure, µg/m^3^	23.73 (19.61–27.29)	23.08 (19.81–27.03)	23.73 (19.61–27.29)	0.64
PM_2.5_ exposure, µg/m^3^	12.19 (10.50–14.98)	11.98 (10.36–15.02)	12.19 (10.56–14.98)	0.57
SO_2_ exposure, µg/m^3^	3.78 (2.83–5.47)	3.61 (2.69–5.44)	3.80 (2.90–5.47)	0.06
CO exposure, µg/m^3^	0.23 (0.19–0.30)	0.23 (0.19–0.30)	0.22 (0.19–0.30)	0.95
NO_2_ exposure, µg/m^3^	16.20 (12.16–24.47)	15.47 (10.81–22.31)	16.39 (12.47–24.63)	*p* < 0.01

^1^ Data are presented as median (interquartile range) or N (% subjects). Differences across SP-D categories were measured by ANOVA adjusted by age, sex and BMI or chi-squared test. BMI: body mass index; HOMA: homeostasis model assessment; HDL: high-density lipoprotein; LDL: low-density lipoprotein; PM_10_: particulate matter < 10 µm; PM_2.5_: particulate matter < 2.5 µm; SO_2_: sulfur dioxide; CO: carbon monoxide; NO_2_: nitrogen dioxide.

**Table 2:** Partial spearman correlations between serum SP-D levels and exposure variables ^2^.

Variable	Rho	*p* Value
Tobacco exposure		
Smoking years	0.17	*p* < 0.001
Cigarettes per day	0.07	0.02
Total cigarettes	0.16	*p* < 0.001
Air pollutants exposure		
PM_10_	0.02	0.24
PM_2.5_	0.02	0.42
SO_2_	0.04	0.07
CO	0.01	0.66
NO_2_	0.05	0.02

^2^ Adjusted by age and sex. PM_10_: particulate matter < 10 µm; PM_2.5_: particulate matter < 2.5 µm; SO_2_: sulfur dioxide; CO: carbon monoxide; NO_2_: nitrogen dioxide.

**Table 3:** Multivariable association analysis between SP-D categories and the risk of incident T2DM after 7.5 years ^3^.

	OR (95% CI)	*p* Value
SP-D (high)	1.85 (1.08–3.15)	0.02
Age	1.04 (1.02–1.06)	*p* < 0.001
Sex (women)	0.81 (0.54–1.20)	0.28
BMI	1.09 (1.04–1.14)	*p* < 0.001
Fasting serum glucose	1.07 (1.05–1.09)	*p* < 0.001
Family history T2DM	1.90 (1.26–2.86)	*p* < 0.01
Insulin resistance	1.30 (0.83–2.03)	0.25
Smoking status (current smokers)	1.62 (1.01–2.61)	0.04

^3^ Adjusted by potential confounders for T2DM: age, sex, BMI, fasting serum glucose, family history of T2DM, insulin resistance and smoking status.

## Data Availability

The data that support the findings of this study are available from the corresponding author upon reasonable request. The data are not publicly available as it is part of an ongoing study.

## Abbreviations

The following abbreviations are used in this manuscript:

T2DM	Type 2 Diabetes Mellitus
SP-D	Surfactant Protein D
HOMA	Homeostasis Model Assessment
BMI	Body Mass Index
LDL	Low Density Lipoproteins
HDL	High Density Lipoproteins
PM_10_	Particle Matter < 10µ
PM_2.5_	Particle Matter < 2.5μ
SO_2_	Sulfur Dioxide
CO	Carbon Monoxide
NO_2_	Nitrogen Dioxide
